# Tonsillectomy and the incidence of various types of cancer

**DOI:** 10.1007/s12026-021-09230-3

**Published:** 2021-09-15

**Authors:** Gábor Holló

**Affiliations:** Institute of Psychology, University of Debrecen, Egyetem tér 1., 4032 Debrecen, Hungary

**Keywords:** Tonsillectomy, Cancer, T cells, NK cells

## Abstract

A potential connection between tonsillectomy and the development of various cancer types has repeatedly been reported in the scientific literature, but many studies have contradicted these observations. Thus, we have no clear evidence, neither to firmly support nor to refute the above-mentioned connection. Here, I suggest that the main reason for the lack of clearer evidence is that the investigations have so far mainly used incorrect sample groups. I propose that individual differences in the tonsils’ involvement in immune reactions should be taken into account to solve this long-standing puzzle.

The hypothesis that tonsillectomy increases the chance of tumour formation has been a regularly emerging theme in the medical literature for decades. It has been suggested that tonsillectomy could promote the emergence of various cancer types. It was found, for example, that tonsillectomy increased the liability to Hodgkin lymphoma [1], and the same conclusion has also been drawn by subsequent studies [2, 3]. A positive relationship between leukaemia and tonsillectomy was also observed in the early 70 s [4] and also repeatedly reported in later decades [5, 6]. An increasing effect of tonsillectomy on breast cancer risk with increasing age has also been shown [7]. It was proposed that tonsillectomy increased the risk of breast cancer in different target groups [8, 9]. It was also presented that a history of tonsillectomy correlated with a greater risk of prostate cancer [10]. A Taiwanese nationwide retrospective cohort study reported that a higher overall risk of all cancer types and a marginally significantly increased risk of breast cancer development was observed in tonsillectomised patients with more than 3 years of follow-up [11]. Tonsillectomy was also associated with heightened odds of developing base of tongue cancer [12].

However, other studies have reported results different from these. For example, it has been concluded that tonsillectomy did not affect carcinogenesis [13] and that there was no significant relationship between tonsillectomy and acute leukaemia [14]. Likewise, the frequency of tonsillectomy was shown to be greater in the control group than in the examined cancerous groups (e.g. [15]). It was also suggested that a higher proportion of tonsillectomy correlated with a decrease in Hodgkin lymphoma risk [16] and pancreatic cancer risk [17]. It was reported that tonsillectomy decreased the odds of tonsil cancer [12]. Some studies with small sample sizes found that the initial immunological deficit following tonsillectomy in children becomes normal after 1 month [18], 6 months [19], and 54 months [20]. Similarly, a recent study concluded that the examined humoral and cellular immunity markers were not altered by adenoidectomy with/without tonsillectomy in children, nor did the surgery lead to an increased risk of infection, nor to immune-deficiency disorders in the 3 months following the operation [21].

On the other hand, it has been emphasised that it was possible to develop cancer due to factors underlying tonsillectomy, such as tonsillitis, rather than to tonsillectomy itself [3]. Similarly, another study also pointed out that susceptibility to infections could explain both the formation of cancer and the necessity of the removal of the lymphatic tissue [5].

It is important to underline, however, that all the studies which were carried out on a considerably large sample (*N*_tot_ ≥ roughly 5000) reported some sort of positive relationship between tonsillectomy and cancer [2, 3, 10, 11].

On the basis of these results, the picture which emerges of the possible relationship between tonsillectomy and cancers is obscure and full of contradictions.

To explain this seemingly embarrassing pattern which unfolds from the literature, it may well be useful to take a closer look at the population samples and control groups used by the different studies. These groups vary on a broad spectrum, both in terms of number and composition; however, they share a common feature, namely that they do not take into account the potential individual differences regarding the *involvement of the tonsils in immunity*. As this argument has not still been investigated, it needs subpopulational analyses to determine how the presumptive differences manifest and to what extent.

It is a well-known phenomenon among otolaryngologists that the immunological functions of the tonsils are rich in childhood but decline as children become adults (e.g. [22–25]). Perhaps the best sign of this is that the majority of children have large, hypertrophic tonsils, while only a minority of adults do. The tonsils are also subject to substantial age-related changes in their cellular composition [26]. Nevertheless, the immune functions of adult tonsils are also important and manifold [27–29].

The role of the tonsils as secondary lymphoid organs in both humoral and cellular immunological processes has been known for a long time (e.g. [22–25]). However, it is important to highlight that in the past decade, significant progress has been made regarding the functions of the tonsils in immunity. In addition to their traditionally known functions of harbouring and maturing lymphoid cells, it has become evident that the tonsils are also capable of generating a subset of these cells. For example, it has been reported that developing T cell precursors were detected extrathymically in the tonsils [30]: the tonsils actively produce T cells due to their comprehensive T cell development programme. In addition to producing and maturing T cells involved in adaptive immunity, the tonsils’ contribution to the development of another subset of immune cells with a potent anti-tumoral activity, the natural killer (NK) cells, which are part of innate immunity, has also received strong support [31–33].

If T and NK cell development and maturation occurs in the tonsils, then tonsillectomy probably has consequences on T and NK cell functioning. Since both T cells and NK cells play an important role in the fight against tumours, tumorigenesis could logically—at least in certain cases and to a certain degree—be affected by the removal of the tonsils.

The tonsils may contribute to immune cell development differently in different people. Large tonsils in adulthood may be more immunologically functional than small tonsils. This notion is in line with a recent study conducted in children [34], although the phenomenon is far from being explored satisfactorily. However, as mentioned above, the hypertrophic tonsils may also be indicators of increased susceptibility to infections, and it could be these infections which may lead both to tonsillectomy and progress to various cancers [3, 5], especially when the tonsils are removed. In this regard, enlarged tonsils mas also indicate individuals fighting against more dangerous and potentially oncogenic infections, so the size of the tonsils could also simply reflect more dangerous and less dangerous infections. In summary, large tonsils could be indicative both of a greater threat from oncogenic pathogens, and of a high importance of the tonsils in immunologic activity.

Naturally, the degree of involvement of the tonsils in the immune responses may also not be connected to their size. Molecular and/or cellular markers are likely to be more reliable features in indicating their level of participation in immunity. Retinoic acid–inducible gene I (RIG-I), interferon alpha (IFN-α), mitochondrial antiviral-signaling protein (MAVS), NLR family pyrin domain containing 3 (NLRP3), toll-like receptor (TLR) 4 and TLR7, and several inflammatory markers (such as IL-1β, NF-κB, and IL-7) have been shown to be highly expressed in simple hypertrophic tonsils (i.e. not in hypertrophic tonsils with recurrent tonsillitis), showing the activity of pathogen-induced innate immune responses [34]. Likewise, neutrophil–lymphocyte ratio (NLR), lymphocyte-monocyte ratio (LMR), platelet-lymphocyte ratio (PLR), and mean platelet volume (MPV) have been determined as indicators of systemic inflammation in adults [e.g. 35]. More specifically, the values of NLR [35–37], PLR [36], and LMR [38–40] have also been used for the prognosis of different types of cancer. However, the precise molecular and/or cellular indicators of the tonsils’ immunologic activity are still to be determined.

From all this, it follows that childhood tonsillectomy will probably negatively affect those people who would have highly functional tonsils in adulthood, but not those whose tonsils would gradually lose their importance in immunity as the individual grows.

Thus, if the study samples examined were properly selected, a possible solution to the puzzle represented by tonsillectomy and its potential relation to cancer could be found. So far, both the tonsillectomised and the control groups of the studies cited have always also included individuals with presumably low functioning tonsils, which has probably biased the results by masking the pattern; i.e., it has shown tonsillectomy to be less harmful than it could be for sensitive people. On the one hand, the size of study samples may significantly influence the appearance of the real pattern: larger samples are expected to unveil it better [2, 3, 10, 11]. On the other hand, the composition of the samples is also important: tonsillectomy tends to be less likely in individuals with small tonsils, who may therefore be more heavily represented in the control group than in the tonsillectomy group.

A much more accurate procedure would be to look for adults with highly functional tonsils in the first place, to identify—if possible—molecular and/or cellular markers which differentiate them from those with low functioning tonsils, and to see whether the pattern holds when creating both the tonsillectomised group and the control group only from individuals with highly functioning tonsils who are potentially vulnerable to tonsillectomy. With the help of this approach, vulnerable children could also be identified, helping doctors in making decisions about childhood tonsillectomy.

Tonsillectomy has been reported to potentially cause serious—sometimes fatal—consequences (e.g. [41–43]). In addition to this risk, the potentially impaired—or conversely, untouched—immune functions should also be considered while planning a personalised medical intervention to the tonsils.

## Data Availability

Not applicable.

## References

[1] Vianna NJ, Greenwald P, Davies JNP (1971). Tonsillectomy and Hodgkin’s disease: the lymphoid tissue barrier. Lancet.

[2] Liaw K-L, Adami J, Gridley G, Nyren O, Linet MS (1997). Risk of Hodgkin’s disease subsequent to tonsillectomy: a population-based cohort study in Sweden. Int J Cancer.

[3] Vestergaard H, Westergaard T, Wohlfahrt J, Hjalgrim H, Melbye M (2010). Tonsillitis, tonsillectomy and Hodgkin’s lymphoma. Int J Cancer.

[4] Cuneo JM (1972). Tonsillectomy and leukaemia. Lancet.

[5] Schüz J, Kaletsch U, Meinert R, Kaatsch P, Michaelis J (1999). Association of childhood leukaemia with factors related to the immune system. Br J Cancer.

[6] Vineis P, Miligi L, Crosignani P, Davico L, Fontana A, Masala G, Nanni O, Ramazzotti V, Rodella S, Stagnaro E, Tumino R, Viganò C, Vindigni C, Costantini AS (2003). Delayed infection, late tonsillectomy or adenoidectomy and adult leukaemia: a case–control study. Br J Cancer.

[7] Lubin JH, Burns PE, Blot WJ, Lees AW, May C, Morris LE, Fraumeni JF Jr (1982). Risk factors for breast cancer in women in northern Alberta, Canada, as related to age at diagnosis. J Natl Cancer Inst..

[8] Yasui Y, Potter JD, Stanford JL, Rossing MA, Winget MD, Bronner M, Daling J (2001). Breast cancer risk and “delayed” primary Epstein-Barr virus infection. Cancer Epidemiol Biomarkers Prev.

[9] Brasky TM, Bonner MR, Dorn J, Marshall JR, Vena JE, Brasure JR, Freudenheim JL (2009). Tonsillectomy and breast cancer risk in the Western New York Diet Study. Cancer Causes Control.

[10] Whittemore AS, Paffenbarger RS, Anderson K, Lee JE (1985). Early precursors of site-specific cancers in college men and women. J Natl Cancer Inst.

[11] Sun L-M, Chen H-J, Li T-C, Sung F-C, Kao C-H (2014). A nationwide population-based cohort study on tonsillectomy and subsequent cancer incidence. Laryngoscope.

[12] Zevallos JP, Mazul AL, Rodriguez N, Weissler MC, Brennan P, Anantharaman D, Abedi-Ardekani B, Hayes DN, Olshan AF (2016). Previous tonsillectomy modifies odds of tonsil and base of tongue cancer. Br J Cancer.

[13] Gross L (1966). Incidence of appendectomies and tonsillectomies in cancer patients. Cancer.

[14] Freeman AI, Lieberman N, Tidings J, Bross I, Glidewell O (1971). Previous tonsillectomy and the incidence of acute leukaemia of childhood. Lancet.

[15] Cassimos C, Sklavunu-Zurukzoglu S, Catriu D, Panajiotidu C (1973). The frequency of tonsillectomy and appendectomy in cancer patients. Cancer.

[16] Bonelli L, Vitale V, Bistolfi F, Landucci M, Bruzzi P (1990). Hodgkin’s disease in adults: association with social factors and age at tonsillectomy. A case-control study Int J Cancer.

[17] Zhang J, Prizment AE, Dhakal IB, Anderson KE (2014). Cholecystectomy, gallstones, tonsillectomy, and pancreatic cancer risk: a population-based case-control study in Minnesota. Br J Cancer.

[18] Kaygusuz I, Gödekmerdan A, Karlidag T, Keles E, Yalçin S, Aral I, Yildiz M (2003). Early stage impacts of tonsillectomy on immune functions of children. Int J Pediatr Otorhinolaryngol.

[19] Zielnik-Jurkiewicz B, Jurkiewicz D (2002). Implication of immunological abnormalities after adenotonsillotomy. Int J Pediatr Otorhinolaryngol.

[20] Kaygusuz I, Alpay HC, Gödekmerdan A, Karlidag T, Keles E, Yalçin S, Demir N (2009). Evaluation of long-term impacts of tonsillectomy on immune functions of children: a follow-up study. Int J Pediatr Otorhinolaryngol.

[21] Yan Y, Song Y, Liu Y, Su J, Cui L, Wang J, Geng J, Liu X, Shi Y, Quan S, Hang A, Zuo L (2019). Short- and long-term impacts of adenoidectomy with/without tonsillectomy on immune function of young children <3 years of age. Medicine (Baltimore).

[22] Brandtzaeg P, Bernstein J, Ogra P (1987). Immune functions and immunopathology of palatine and nasopharyngeal tonsils. Immunology of the ear.

[23] Andersson J, Abrams J, Björk L, Funa K, Litton M, Agren K, Andersson U (1994). Concomitant in vivo production of 19 different cytokines in human tonsils. Immunology.

[24] Bernstein JM, Gorfien J, Brandtzaeg P, Ogra P (1999). The immunobiology of the tonsils and adenoids. Mucosal immunology.

[25] Nave H, Gebert A, Pabst R (2001). Morphology and immunology of the human palatine tonsil. Anat Embryol.

[26] Bergler W, Adam S, Gross HJ, Hormann K, Schwartz AR (1999). Age-dependent altered proportions in subpopulations of tonsillar lymphocytes. Clin Exp Immunol.

[27] Weigel C, Geissler K, Markwart R, Schubert K, Rubio I, Guntinas-Lichius O, Requardt RP (2015). Isolation of viable and functional T-cells from human palatine tonsils. J Immunol Methods..

[28] Geißler K, Markwart R, Requardt RP, Weigel C, Schubert K, Scherag A, Rubio I, Guntinas-Lichius O (2017). Functional characterization of T-cells from palatine tonsils in patients with chronic tonsillitis. PLoS ONE.

[29] Geißler K, Weigel C, Schubert K, Rubio I, Guntinas-Lichius O (2020). Cytokine production in patients with recurrent acute tonsillitis: analysis of tonsil samples and blood. Sci Rep.

[30] McClory S, Hughes T, Freud AG, Briercheck EL, Martin C, Trimboli AJ, Yu J, Zhang X, Leone G, Nuovo G, Caligiuri MA (2012). Evidence for a stepwise program of extrathymic T cell development within the human tonsil. J Clin Invest.

[31] Freud AG, Yu J, Caligiuri MA (2014). Human natural killer cell development in secondary lymphoid tissues. Semin Immunol.

[32] Scoville SD, Mundy-Bosse BL, Zhang MH, Chen L, Zhang X, Keller KA, Hughes T, Chen L, Cheng S, Bergin SM, Mao HC, McClory S, Yu J, Carson WE, Caligiuri MA, Freud AG (2016). A progenitor cell expressing transcription factor RORgammat generates all human innate lymphoid cell subsets. Immunity.

[33] Scoville SD, Freud AG, Caligiuri MA (2017). Modeling human natural killer cell development in the era of innate lymphoid cells. Front Immunol.

[34] Huang Q, Hua H, Li W, Chen X, Cheng L (2020). Simple hypertrophic tonsils have more active innate immune and inflammatory responses than hypertrophic tonsils with recurrent inflammation in children. J Otolaryngol-Head N.

[35] Absenger G, Szkandera J, Pichler M, Stotz M, Arminger F, Weissmueller M, Schaberl-Moser R, Samonigg H, Stojakovic T, Gerger A (2013). A derived neutrophil to lymphocyte ratio predicts clinical outcome in stage II and III colon cancer patients. Br J Cancer.

[36] Wang D, Yang J-X, Cao D-Y, Wan X-R, Feng F-Z, Huang H-F, Shen K, Xiang Y (2013). Preoperative neutrophil-lymphocyte and platelet-lymphocyte ratios as independent predictors of cervical stromal involvement in surgically treated endometrioid adenocarcinoma. Onco Targets Ther.

[37] Chen J, Hong D, Zhai Y, Shen P (2015). Meta-analysis of associations between neutrophil-to-lymphocyte ratio and prognosis of gastric cancer. World J Surg Oncol.

[38] Hu P, Shen H, Wang G, Zhang P, Liu Q, Du J (2014). Prognostic significance of systemic inflammation-based lymphocyte- monocyte ratio in patients with lung cancer: based on a large cohort study. PLoS ONE.

[39] Stotz M, Pichler M, Absenger G, Szkandera J, Arminger F, Schaberl-Moser R, Samonigg H, Stojakovic T, Gerger A (2014). The preoperative lymphocyte to monocyte ratio predicts clinical outcome in patients with stage III colon cancer. Br J Cancer.

[40] Han L-H, Jia Y-B, Song Q-X, Wang J-B, Wang N-N, Cheng Y-F (2015). Prognostic significance of preoperative lymphocyte-monocyte ratio in patients with resectable esophageal squamous cell carcinoma. Asian Pac J Cancer Prev.

[41] Windfuhr JP, Schlöndorff G, Baburi D, Kremer B (2008). Serious posttonsillectomy hemorrhage with and without lethal outcome in children and adolescents. Int J Pediatr Otorhinolaryngol.

[42] Windfuhr JP, Schlöndorff G, Sesterhenn AM, Kremer B (2009). From the expert’s office: localized neural lesions following tonsillectomy. Eur Arch Otorhinolaryngol.

[43] Windfuhr JP, Schlöndorff G, Sesterhenn AM, Prescher A, Kremer B (2009). A devastating outcome after adenoidectomy and tonsillectomy: ideas for improved prevention and management. Otolaryngol Head Neck Surg.

[44] Lee JS, Kim NY, Na SH, Youn YH, Shin CS (2018). Reference values of neutrophil-lymphocyte ratio, lymphocyte-monocyte ratio, platelet-lymphocyte ratio, and mean platelet volume in healthy adults in South Korea. Medicine..

